# Hardhat-Wearing Detection Based on a Lightweight Convolutional Neural Network with Multi-Scale Features and a Top-Down Module

**DOI:** 10.3390/s20071868

**Published:** 2020-03-27

**Authors:** Lu Wang, Liangbin Xie, Peiyu Yang, Qingxu Deng, Shuo Du, Lisheng Xu

**Affiliations:** 1 School of Computer Science and Engineering, Northeastern University, Shenyang 110016, China; wanglu@cse.neu.edu.cn (L.W.); xlb_neu@163.com (L.X.); 1771472@stu.neu.edu.cn (P.Y.); dengqx@mail.neu.edu.cn (Q.D.); 2 College of Medicine and Biological Information Engineering, Northeastern University, Shenyang 110016, China; ds956688@163.com; 3 The Key Laboratory of Intelligent Computing in Medical Image, Ministry of Education, Northeastern University, Shenyang 110016, China

**Keywords:** hardhat-wearing detection, convolutional neural network, real-time detection

## Abstract

Construction sites are dangerous due to the complex interaction of workers with equipment, building materials, vehicles, etc. As a kind of protective gear, hardhats are crucial for the safety of people on construction sites. Therefore, it is necessary for administrators to identify the people that do not wear hardhats and send out alarms to them. As manual inspection is labor-intensive and expensive, it is ideal to handle this issue by a real-time automatic detector. As such, in this paper, we present an end-to-end convolutional neural network to solve the problem of detecting if workers are wearing hardhats. The proposed method focuses on localizing a person’s head and deciding whether they are wearing a hardhat. The MobileNet model is employed as the backbone network, which allows the detector to run in real time. A top-down module is leveraged to enhance the feature-extraction process. Finally, heads with and without hardhats are detected on multi-scale features using a residual-block-based prediction module. Experimental results on a dataset that we have established show that the proposed method could produce an average precision of 87.4%/89.4% at 62 frames per second for detecting people without/with a hardhat worn on the head.

## 1. Introduction

Construction sites are some of the most dangerous places and are fraught with risks, which reports tens of thousands of injuries and deaths throughout the world every year [1]. Many such injuries and deaths can be alleviated or even avoided if the workers wear safety hardhats, as hardhats can protect workers by resisting penetration by objects, absorbing shock from blows to the head, and reducing electrical shock hazards in accidents on construction sites [2]. However, workers may occasionally forget to wear hardhats or simply be unwilling to wear them due to the inconvenience or the discomfort. Therefore, supervision of workers’ hardhat use on construction sites is needed. However, as manual inspection is labor-intensive and expensive, methods for automatically inspecting if people have hardhats on their heads are desired on modern construction sites.

In the past few years, some efforts have been made by researchers to solve the hardhat-wearing detection problem based on traditional computer vision and machine-learning techniques [1,3,4,5,6,7]. Most of these methods employ multi-step strategies. Specifically, they usually leverage background subtraction to extract moving targets first. Then, person detection is performed to obtain the approximate positions and sizes of people in the images. Finally, head regions are estimated, within which the hardhat detectors are applied to check if hardhats exist. However, these methods have some obvious drawbacks. First, for people without obvious motion, background subtraction would fail to extract the image regions corresponding to people. This is likely to happen when workers stay at a place to work on something. Second, occlusion happens frequently on construction sites, which may lead to inaccurate person detection and consequently negatively affect the following hardhat detection. Third, workers may exhibit various postures while working, but people detectors are mostly trained to detect standing/walking people. This discrepancy would also degrade the people-detection accuracy. Moreover, as the overall frameworks of existing methods consist of multiple stages, they are not easy to deploy in real applications.

Recently, with the prevalence of deep learning, researchers have attempted to apply convolutional neural networks (CNNs) for object detection and obtained impressive results [8,9,10]. However, the research on applying deep learning to hardhat detection is still in its infancy and there are few such works published to date. Fang et al. [2] propose handling the hardhat detection problem with deep learning, using a Faster R-CNN network [10]. Due to the automatic feature-extraction capability and high discrimination power of CNN networks, the approach in [2] exhibits several advantages over traditional approaches: (1) It does not have to perform background subtraction or full-body person detection, meaning that it is not limited to detecting moving persons, nor does it require that the persons are mostly visible. (2) It can produce good performance when people exhibit various postures (e.g., standing, bending, sitting, and squatting). Therefore, it can be applied in complex and unconstrained scenarios. (3) As the Faster R-CNN network can be trained end-to-end, this detector is much easier to deploy than traditional multi-step hardhat detection methods. Nevertheless, Fang et al. [2] directly employ the ready-made Faster R-CNN network for hardhat detection without performing any modification to adapt it to the specific task. The problem with Faster R-CNN is that as a complex two-stage detection network originally developed for detecting objects of several tens of classes, Faster R-CNN is somewhat overkill for hardhat-wearing detection. In addition, the large number of parameters of the Faster R-CNN model prevents it from being implemented in embedded systems. Recently, Wu et al. proposed the use of the Single Shot MultiBox Detector (SSD) [9] with reverse progressive attention for hardhat-wearing detection [11]. This method can deal with the hardhat-wearing detection problem better than some classical detection networks. However, the model size of the network is still very large.

As such, in this paper, we propose a lightweight network designed specifically for detecting if people are wearing hardhats. Specifically, the proposed detection network uses the efficient MobileNet model [12] as the backbone to extract the basic multi-scale feature maps. After the backbone network, a top-down module is attached to combine low-level and high-level CNN features. As the filters in higher convolutional layers tend to capture the overall semantic information of an object as well as the contextual information, while the high spatial resolution is kept in lower layers, merging the features from both higher and lower layers can enhance the detection performance of the network on small and occluded targets. As to the prediction module, in addition to the traditional classification and regression layers, a residual block [13] is added before them in our method to extract stronger features that are more suitable for classification and bounding box regression. Predictions are finally made on the multi-scale features respectively to detect target objects of different sizes.

To obtain an effective CNN model, the proposed network is trained and tested with a hardhat-wearing detection dataset that we have collected and annotated. With the concise one-step detection framework and the efficient network design, the proposed method enables high-speed hardhat-wearing detection at 62 frames per second (FPS), which is much faster than most existing methods (∼10 FPS). Compared with Faster R-CNN, our method also runs faster (∼5×) and achieves higher detection accuracy (∼1.7%) with a much smaller model size (18.7 MB vs. 607.2 MB).

In summary, the contributions we made in this paper are as follows:

- An end-to-end trainable CNN model for hardhat-wearing detection is proposed, in which the MobileNet backbone, the top-down module and the residual-block-based prediction module work together to ensure fast and robust no_hardhat and hardhat detection;
- A hardhat-wearing detection dataset is introduced for the training and testing of hardhat detection approaches, which has been made publicly available;
- Average precisions of 87.4% and 89.4% for detecting people without and with a hardhat worn on the head was achieved by the proposed detector, at the real-time running speed of 62 FPS.

For concise presentation, in the rest of this paper, the terms “hardhat” and “no_hardhat” are used to represent the people with and without hardhats on their heads, respectively.

## 2. Related Works

In this section, hardhat/no_hardhat detection approaches using hand-crafted features and traditional classifiers are first summarized. Then, object detection works based on deep CNNs are reviewed.

### 2.1. Hardhat/No_hardhat Detection Using Traditional Methods

Since the circle-like shape of heads and hardhats is very common, it is not easy to build a reliable hardhat-wearing detector using hand-crafted features, due to their limited representation power. In addition, heads and hardhats often appear as small objects in images, which further increases the detection difficulty. Therefore, existing methods typically solve the hardhat-wearing detection problem with three steps, with the aim of increasing the robustness of the detectors. The three steps are: (a) moving object segmentation, (b) full-body detection, and (c) hardhat/no_hardhat classification or detection.

The first step is to segment the moving object, which can reduce the computational cost of the following target detection processes (e.g., full-body and hardhat detection), as only the area with motion needs to be analyzed. Extracting the moving objects can also avoid false-positive detections from the background. For example, Wu et al. [7] use KNN for background subtraction, while Li et al. [6] apply the ViBe method [14] to segment the moving objects. However, by extracting the objects with motion, targets that are not moving cannot be detected. This is a great limitation for the application of hardhat detection on construction sites, as not all the workers are expected to move while working.

As human body shape is more discriminative than the circular-like shape of heads and hardhats, full-body person detection is usually adopted as the second step to assist the hardhat/no_hardhat localization [1,4,6,15]. For example, Gualdi et al. [4] apply the covariance descriptors and the LogitBoost classifier for people detection. Park et al. [1] and Wu et al. [7] use the classical combination of histogram of oriented gradient (HOG) feature [16] and support vector machine (SVM) to perform people detection. Recently, Li et al. [6] applied a dedicated people-detection method C4 [17] to detect humans, which extracts a kind of contour features called CENTRIST and uses the more efficient cascaded SVM for classification. However, people detection is prone to fail when human bodies show large deformations (e.g., bending and sitting). The performance of people detection also degrades drastically when occlusion occurs.

Once the person is detected, location of the head can be roughly estimated, and then hardhat detection can be made. From the prior knowledge that safety hardhats worn in dangerous workplaces are usually of some specific colors (e.g., red, yellow, blue, and white), some methods extract color features from the RGB, HSV, and Lab color space [4,5,6,15] to identify hardhats. The shape of hardhats is also exploited for hardhat detection. Considering the good shape description capability of the HOG feature, Park et al. [1] apply the same HOG and SVM combination as is used in the human detection for hardhat detection. To increase the representation power of features, Jia et al. [5] concatenate multiple types of features to form hybrid descriptors for hardhat detection. These features include the HOG feature, the block-based local binary patten feature and some color features. Then, the deformable part model (DPM) is applied to detect hardhats. The advantage of DPM is that its part-based nature enables it to deal with occlusion and it can also model different viewing angles with multiple components. Similarly, Wu et al. [7] propose the use of a hybrid descriptor that consists of LBP, Hu moment invariants, and color histogram for hardhat identification. With the person detection and hardhat detection results, if a hardhat cannot be found within a reasonable area of a person, that person is considered to be not wearing a hardhat.

### 2.2. Object Detection Using Convolutional Neural Networks

CNNs have been applied to a variety of computer vision tasks, such as image classification [18], object detection [10], semantic segmentation [19], facial recognition [20], and hyperspectral image recognition [21], among others. Specifically, in the object detection field, networks such as Faster R-CNN [10], R-FCN [8], YOLO [22], and SSD [9] have achieved high accuracy and hence attracted a great deal of attention from researchers. Faster R-CNN and R-FCN are two-stage networks consisting of the region proposal generation and proposal classification processes. In contrast, YOLO and SSD are single feed-forward convolutional networks that directly predict object bounding boxes and their classes, and thus run very quickly.

With regards to the hardhat-wearing detection task, CNNs have not yet been widely applied. Fang et al. [2] proposed the directly application of the Faster R-CNN network [10] to deal with the hardhat-wearing detection problem. In this method, construction workers that do not wear hardhats are annotated on images for training and testing. The method achieved high accuracy, with both the precision and recall rates greater than 90%. Wu et al. proposed the use of the SSD with reverse progressive attention for hardhat-wearing detection [11]. The reverse progressive attention module can encode multi-level contextual information to generate more abstract features for hardhat-wearing detection and hence performs better than the original SSD. There is also one work that exploits the use of a CNN for helmet/non-helmet detection in a traffic scene [23]. In this work, Vishnu et al. applied the classical three-step framework employed in traditional hardhat-wearing detection approaches for safety helmet-wearing detection, and CNNs were applied for motorcyclist detection and helmet/no_helmet classification. Compared to the HOG-SVM-based detectors, the CNN-based detectors improved the motorcyclist and helmet detection accuracy by 10.0% and 29.3% respectively, demonstrating the capability of CNNs to handle the helmet-wearing detection task.

In this paper, we present a lightweight, end-to-end trainable network for the hardhat-wearing detection task. This method is more practical than existing multi-step hardhat-wearing detectors, as it does not make any additional assumptions on people’s occlusion or posture status, it is easy to train, and it runs in real time with high detection accuracy. Compared to the Faster R-CNN and SSD schemes, our method runs much faster and achieves a competitive accuracy.

## 3. Proposed Method

The aim of this work is to detect people that are not wearing safety hardhats so that alarms can be sent to the administrators. Toward this end, instead of training a detector that can only detect heads not wearing hardhats, both classes (i.e., heads with and without hardhats) are trained and detected. By doing so, the difference between heads with and without hardhats can be learned by the network and hence a robust performance of the detector can be expected.

**Figure 1:**
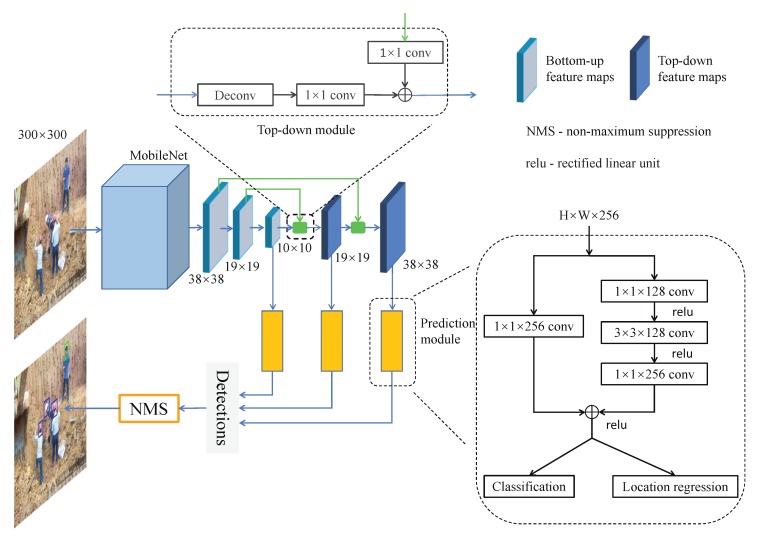
Architecture of the proposed network for detecting and classifying no_hardhat and hardhat instances.

Figure 1 illustrates the architecture of the proposed network. It consists of three core components: the MobileNet as the backbone network to extract multi-scale feature maps, the top-down module to fuse shallower layer features with deeper layer ones, and the residual-block-based prediction module for no_hardhat and hardhat classification and bounding box regression. In the following, each component of the network will be described in detail.

### 3.1. Backbone Network—MobileNet

To achieve real-time object detection, the MobileNet [12] is chosen as the backbone network to extract preliminary features. The MobileNet model is based on depthwise separable convolution, which factorizes a standard convolution into a depthwise convolution and a 1×1 convolution called pointwise convolution. The depthwise convolution applies a single filter to each input channel and then the pointwise convolution applies a 1×1 convolution to combine the outputs of the depthwise convolution.

In the proposed method, the outputs of the last three convolutional layers of the MobileNet backbone are used as the input to the following top-down module.

### 3.2. Top-Down Module

Heads and hardhats often appear in images as small objects, which is a well-known challenging issue in object detection. Small objects are usually detected in feature maps generated by lower convolutional layers of the backbone network, as high spatial resolution is required for small object localization. However, the semantic information contained in lower layers is weak, meaning detection based on these layers is not reliable. On the other hand, the higher convolutional layers generate low-resolution but semantically strong features, which could benefit small-object detection. Therefore, combining the higher- and lower-layer features for object prediction would improve the detection accuracy. In addition, as the higher convolutional layers have larger perception fields, the corresponding feature maps contain extra contextual information to help discriminate the detection ambiguities caused by small objects, occlusion, or uncommon viewing angles. As such, a top-down module is constructed and appended after the backbone network, as shown in Figure 1.

Overall, the top-down module combines the upsampled feature map from a higher layer with the feature map of the same resolution from the lower layer and then outputs the merged feature map. Specifically, the deconvolutional layer upsamples the feature map from the higher layer by a factor of 2 based on the bilinear interpolation algorithm. Then, a 1×1 convolutional block is followed to allow learnable interactions of cross-channel features. After that, the upsampled feature map is merged with the feature map of identical size from the bottom-up convolutional block (which undergoes a 1×1 convolutional block to reduce channel dimension) by elementwise addition.

### 3.3. Prediction Module

The prediction module of the detection network estimates the probabilities of each default box containing an instance of no_hardhat and hardhat. Meanwhile, it regresses the offset for each such box to a nearby ground-truth object, if one exists. As mentioned above, through the top-down module, the feature maps generated from the lower and higher convolutional layers are fused. However, the merged feature maps cannot fit the function of predicting the offsets and the confidence scores of default boxes well due to the over-simplicity of the merging process (i.e., the elementwise addition). To generate better feature representation, a residual block is added before the classification and regression layers in the prediction module for better feature fusion, as shown in Figure 1.

The residual block takes the feature map from the feature pyramid produced by the top-down module as input, and then then forward it into two branches. For the first branch, the 1×1 convolutional block is leveraged to fuse the feature maps across different channels and resolve the vanishing gradient problem by preserving the gradient flow through the entire network. The second branch consists of a bottleneck structure, which consists of a 1×1 convolutional block to reduce the dimension of the feature map, a 3×3 convolutional block to extract deeper features, and another 1×1 convolutional layer to restore the dimension. The outputs of the two branches are then merged through elementwise addition. Compared to a pure 3×3 convolutional block, the bottleneck structure has the advantage of reducing the number of parameters while improving the network performance. A comparison with other possible variants of the residual model is made in Section 4.2.

### 3.4. Training and Loss Functions

The training policy used in this works is similar to that of SSD [9]. The loss function consists of the smooth $L_{1}$ localization loss [24] and the softmax confidence loss. Hard negative mining and data augmentation as suggested in SSD are performed to increase the robustness of the detector.

## 4. Experiment

In this section, the performance of the proposed network on the hardhat-wearing detection task is evaluated on the hardhat-wearing detection dataset constructed by the authors. The detector’s performance on the “no_hardhat” class is more important, as the final aim of this work is to detect people who are not wearing hardhats and then send out alarms to relevant personnel. Regardless, the detector’s performance on the “hardhat” class is also reported, as the hardhat detection result can be used for people detection and tracking, which are needed by construction site surveillance applications. Moreover, by merging the no_hardhat and hardhat detection results, the number of people on construction sites can be obtained. Extensive experiments were also carried out to justify the proposed detector network, including performing an ablation study on the proposed network architecture, replacing the proposed prediction module with other possible variants and comparing the proposed method with existing object detectors.

### 4.1. Dataset

To validate the proposed method, we established a dataset, and it is publicly available at https://doi.org/10.7910/DVN/7CBGOS. Like in the general object detection task, we annotated the head part of each person in the images with a bounding box and an associated label “hardhat” or “no_hardhat”. Our dataset has the following characteristics:

- The backgrounds of the images are diverse, including various construction-site scenes;
- The types of poses exhibited by workers are rich (standing, walking, sitting, bending, squatting, among others);
- People in the images are captured from different viewing angles (e.g., front, back, side, top, and bottom views);
- People are occluded to different degrees, from no occlusion to severe occlusion;
- The images were taken both outdoors and indoors, with different illumination intensities;
- The sizes of heads and hardhats vary significantly, from a few dozen to a few thousand pixels.

The large number of instances and the diversity of the data ensure a reliable CNN model with good performance to be trained while helping to avoid overfitting. There are 7064 images in the dataset; among them, 5298 images were used for training and 1766 for testing. There were a total of 4978 no_hardhat instances and 14,989 hardhat instances in the training set, while the numbers of no_hardhat and hardhat instances in the test set were 1803 and 4863, respectively.

### 4.2. Experimental Setting

All the models in this paper were implemented using the Caffe library [25] and trained on a computer workstation with double Xeon E5-2630 CPUs and the GTX 1080 TI GPU. For better network parameter initialization, the MobileNet backbone was pre-trained for the ImageNet classification task [26]. The input images were resized to 300×300 pixels. The spatial resolutions of the three feature maps for prediction were 38×38, 19×19, and 10×10, respectively. The aspect ratio of the default boxes was set to 0.8. Accordingly, the width and height of the default boxes for each level of feature map were $\sqrt{0.8}s_{k}$ and $s_{k}/\sqrt{0.8}$, respectively, where $s_{k}$ is the scale of the default boxes for the $k^{\mathrm{th}}$ feature map, with $s_{1}=32$, $s_{2}=64$, and $s_{3}=128$, respectively. Stochastic gradient descent [27] was adopted for network training. All models were trained for 40k iterations. The initial learning rate was set to 0.001, which was divided by 10 at the $20k^{\mathrm{th}}$, $30k^{\mathrm{th}}$, and $35k^{\mathrm{th}}$ iterations progressively. A weight decay of 0.005 and a momentum of 0.9 were used. For network inference, Non-Maximum Suppression (NMS) with IoU (the ratio of the overlapping area of the two boxes to the area of their union) 0.45 was applied.

### 4.3. Evaluation Criteria

The detection result is expressed as bounding boxes and the associated detection confidence scores for each image. In an object detection system, the boxes with confidence scores greater than a threshold value are considered to be positives while the others are taken as negatives. The criteria of the PASCAL VOC Challenge [28] are applied in this paper to evaluate the detection results, where precision and recall are calculated as follows:

(1) $$\;Precision=\frac{TP}{TP+FP},$$

(2) $$\;Recall=\frac{TP}{TP+FN},$$

where TP, FP, and FN represent the number of true positive, false positive, and false negative examples, respectively. To determine if a detected box is a true positive or a false positive, the one-to-one correspondence between the detected boxes and ground-truth boxes is first found based on their IoUs. Then, if the IoU between the detected box and the corresponding ground-truth box is greater than 0.5, the detection is considered to be a true positive; otherwise the detection is taken as a false positive. As the performance of a detection algorithm varies with the threshold, the average precision (AP) value was used to evaluate the accuracy of the detection network. In this work, the AP value was computed by averaging the precision at the recall of {0, 0.1, 0.2, …, 1}. In addition, the accuracy metric, calculated as

(3) $$\;Accuracy=\frac{TP}{TP+FP+FN}$$

is used in Section 4.8 to evaluate the performance of different methods under challenging conditions.

### 4.4. Comparison of Different Network Architectures

To demonstrate the effectiveness of the proposed detection network, the performance of three different network architectures was compared. The first was the baseline detection network, which only consists of the MobileNet backbone and the standard prediction module. The second network embeds the top-down module into the baseline network. The third was the proposed network architecture, which further replaces the standard prediction module with the proposed prediction module. The detection results are listed in Table 1. By integrating the top-down module into the baseline network, the AP of the no_hardhat class $AP_{no\_hardhat}$ increased by 2.3%. Further using the proposed prediction module made another 2.3% gain in $AP_{no\_hardhat}$. These results show that both the proposed top-down and prediction modules can promote the network performance in identifying people not wearing hardhats. It can also be seen that the proposed top-down and prediction modules were helpful in improving the hardhat detection performance, with the total gain being 3.4%. Please note that the $AP_{hardhat}$ was always higher than $AP_{no\_hardhat}$ for different network architectures, which might be attributable to two reasons: (1) hardhats are more visually significant and hence easier to be detected than heads, and (2) there are more hardhat samples than no_hardhat samples in our dataset, since all the images were taken on construction sites.

### 4.5. Variants of the Prediction Module

**Figure 2:**
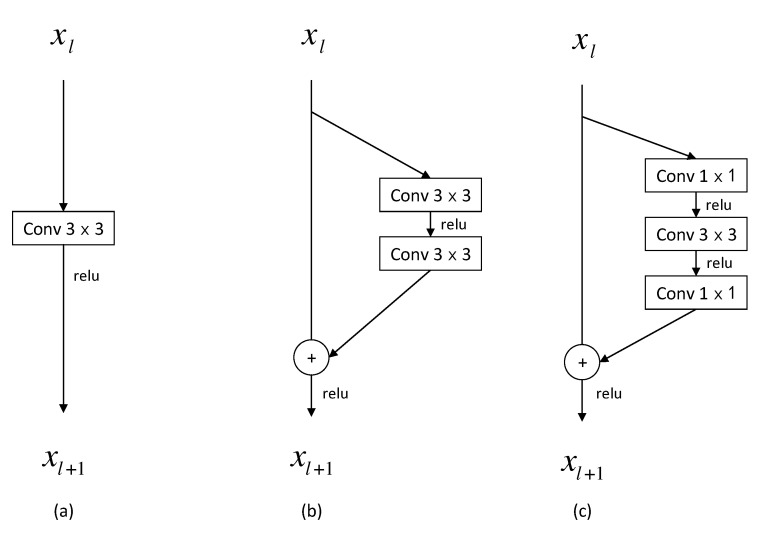
Three variants of the prediction module: (**a**) A 3 × 3 convolutional block; (**b**) A residual block with two 3 × 3 convolutional blocks; (**c**) A residual block with the bottleneck structure.

Three variants of the prediction module, which are the simplified versions of the proposed prediction module, were tested to validate the advantages of the proposed prediction module. The results are listed in Figure 2. Please note that the classification and regression layers are not shown, as they are the same for all the variants of the prediction modules. Variant *a* is a 3×3 convolutional block and variant *b* is a residual block of two branches, with the first branch being a skip connection and the other one consisting of two 3×3 convolutional operations. Variant *c* is also a residual block, with one branch being a skip connection and the other one being a bottleneck structure. For fair comparison, the proposed prediction module was replaced with each of the variants in Figure 2 in our detection network, and the resulting networks were trained with exactly the same optimization scheme. As shown in Table 2, the proposed prediction module resulted in the highest AP for both classes. The residual block, the bottleneck structure, and the cross-channel feature fusion module all promoted the detection performance. In addition, it can be seen that with the bottleneck structure, the model sizes of variant *c* and the proposed prediction module were smaller than those of variant *a* and *b*, due to the 1×1 convolutional operation in the bottleneck structure to reduce the dimension of feature maps.

### 4.6. Comparison with Other Object Detection Methods

We compared the proposed method with two classical general object detection methods (i.e., Faster R-CNN [10] and SSD [9]) on the proposed hardhat-wearing detection dataset. Faster R-CNN is a two-stage detection method, including the object proposal generation stage and the proposal classification and regression stage. The network is large in terms of the total number of parameters and the running speed is relatively low. On the other hand, SSD is a one-stage detection network and it performs classification and regression on pre-defined anchor boxes, instead of the object proposals. SSD runs much faster than Faster R-CNN. The results of these two methods are shown in Table 3. It can be seen that the method proposed in this paper was superior to Faster R-CNN and SSD in terms of running speed and detection accuracy, especially for the no_hardhat class, and our network had a much smaller model size. The results also indicate that as general object detection methods, Faster R-CNN and SSD are too complex and they may result in overfitting for the hardhat-wearing detection task.

### 4.7. Visual Illustration of the Detection Results

In this section, the detection results of the proposed method are illustrated visually. First, we draw a comparison of the results of the three different network architectures mentioned above. Then more examples in which the proposed network gives satisfactory detection results are demonstrated. Finally, the failure cases of the proposed detector are shown, disclosing the limitations that still exist in the proposed method.

The threshold for the detection confidence score was set as the one that produced the minimum total detection error (i.e., the sum of the numbers of false positives and false negatives) for each object class of each network architecture. Specifically, for the no_hardhat class, the threshold was 0.26 for the baseline network, 0.27 for the baseline + top-down network, and 0.25 for the proposed network; whereas for the hardhat class, the three thresholds were 0.31, 0.25, and 0.24, respectively. In each image, the green bounding boxes represent the detected no_hardhat instances while the blue ones denote the hardhat instances.

**Figure 3:**
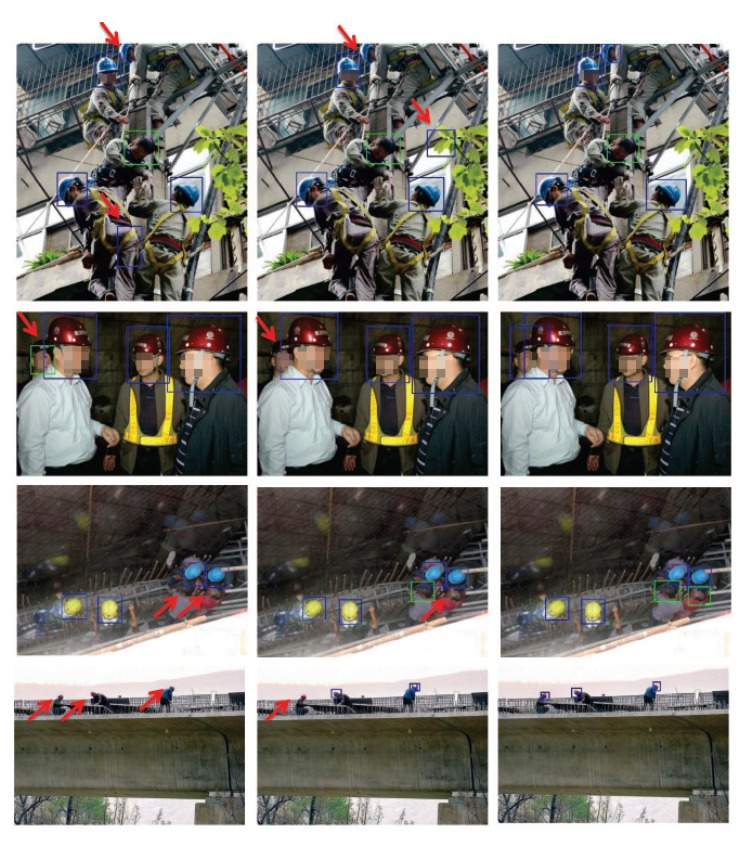
Examples of the detection results using different network architectures. **Left:** baseline; **Middle:** baseline + top-down; **Right:** proposed. Persons indicated by red arrows correspond to incorrect detections.

It can be seen from Figure 3 that the baseline architecture gave the worst performance, while adding the top-down module and using the proposed prediction module progressively improved the detection performance. In the first example, a false alarm hardhat and a missing hardhat were generated by the baseline detector. The missing hardhat is partially out of the image and exhibits an unusual pose. With the top-down module, the false detection was eliminated but another false detection was generated. The proposed network gave the correct detection result, demonstrating its superiority in dealing with both false-negative and false-positive detections. In the second example, an occluded hardhat was either wrongly detected as a no_hardhat instance by the baseline network or missed by the baseline + top-down network, while the proposed architecture was able to correctly detect it. This shows that the proposed method was better able to deal with partial occlusions. In the third example, two no_hardhat instances were missed by the baseline network, as they were taken at an unusual viewing angle and the contrast between the foreground and background is low. Both no_hardhat instances were successfully detected by the proposed detector, proving that the top-down module and the residual block in the prediction module can work synergistically to extract stronger features for the detection task. In the last example, all three low-resolution hardhats were missed by the baseline detector, and the proposed detector could identify all of them. This demonstrates that the proposed method was better able to handle small objects. In summary, the visual comparison of the detection results indicates that the proposed top-down and prediction modules play important roles in improving the detection accuracy of the hardhat-wearing detection network.

**Figure 4:**
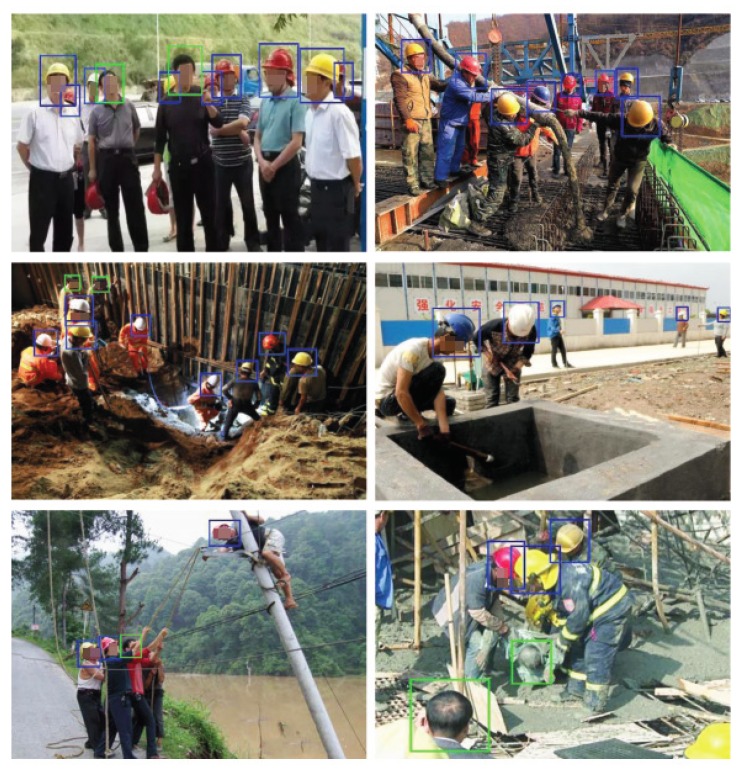
Examples of satisfactory detection results.

Figure 4 displays more examples for which the proposed detector produced satisfactory results. The two images in the first row show that the detector performed well in crowded scenes where occlusion occurred frequently. The two examples in the middle demonstrate that the method performed well in detecting small objects. The two examples in the last row show that the proposed detector was able to detect no_hardhat and hardhat instances taken from different viewing angles. These examples also show that our detector is not sensitive to the deformation of human bodies, which is not true of existing methods based on full-body detection, especially for the case in the bottom-right image where the camera looks downward and the human bodies are basically completely invisible.

**Figure 5:**
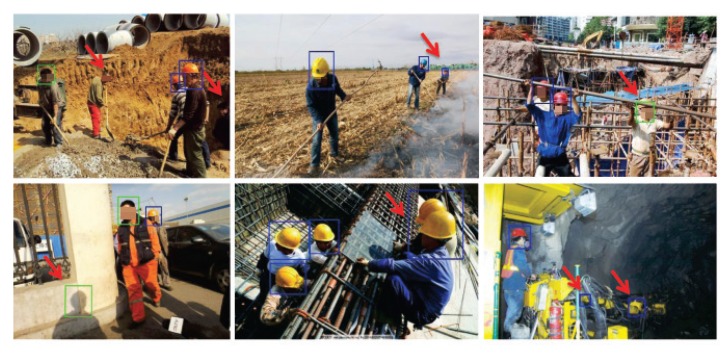
Examples of failure cases.

Although the proposed hardhat-wearing detector exhibits several advantages over existing methods, it is not perfect and might fail in some difficult cases. Figure 5 displays several typical errors made by the proposed method. In the first example, two heads that have low contrast with the background were not detected. As shown in the second and third images, our detector may mistakenly classified a no_hardhat instance as a hardhat instance or vice versa. In the fourth example, the shadow of a head was mistakenly detected as a no_hardhat instance. When there is severe occlusion, as in the case of the fifth image, the proposed method may miss the heavily occluded objects. In the last example, image regions that belong to the construction equipment but with similar color and shape to hardhats were incorrectly detected as hardhats. In general, these errors made by the proposed method are mostly resulting from image ambiguities and could be partly solved if the temporal information could be exploited. Adding more hard negatives is also likely to reduce the errors.

### 4.8. Evaluation of the Proposed Method under Challenging Conditions

In this section, we qualitatively evaluate the performance of our method under challenging occlusion and low-contrast scenarios with the thresholds specified for visual illustration in the last section. Please note that occlusion and low-contrast cases may co-exist with normal cases (i.e., un-occluded and moderate/high-contrast cases) within the same images, especially for the occlusion cases, which makes the calculation of the FP for these situations problematic. To deal with this, in our evaluation, the FP under the occlusion (or low-contrast) condition was calculated by attributing all the false positives in the whole images to occlusion (or low contrast). Though the precision obtained in this way may be lower than the actual value, we are more concerned with the recall values, as occlusion or low-contrast conditions mainly result in missing detections instead of false alarms.

It can be seen from Table 4 and Table 5 that both the occlusion and low-contrast conditions led to low recall rates (less than 80%). Regardless, the proposed method consistently outperformed the baseline and baseline+top-down detectors in terms of the recall rate and the overall accuracy, with large margins (more than 8.5%) for both no_hardhat and hardhat classes in these two scenarios, demonstrating the effectiveness of combining the top-down module with the proposed prediction module for handling difficult cases.

### 4.9. People Counting

Knowing how many people are on a construction site is useful for the construction site management. As a by-product, the proposed detector can be directly applied for people counting by merging the no_hardhat and hardhat detection results. If an object is detected as both no_hardhat and hardhat (e.g., the bounding boxes for the two classes have IoU greater than 0.9), the detection with lower confidence score was discarded. The thresholds for the detection confidence score were set the same as those for the visual illustration. Two metrics were applied to evaluate the detector’s performance in people counting. The first was the error rate (ER), which is computed as:

(4) $$\;ER=\frac{|hc‒\hat{hc}|}{hc}\times 100\%,$$

where hc and $\hat{hc}$ are respectively the total head count from the ground-truth annotations and the total head count produced by the detection network for all the test images. The other metric is the mean square error (MSE), as is computed in the following equation:

(5) $$\;MSE=\frac{1}{N}\times \sum_{i=1}^{N}{\left(hc_{i}‒{\hat{hc}}_{i}\right)}^{2},$$

where *N* is the total number of test images, while $hc_{i}$ and ${\hat{hc}}_{i}$ denote the head counts from the ground truth and the detection network for the $i^{\mathrm{th}}$ test image, respectively. ER considers the total head count in all the images together. However, by doing so, counting errors that occur in different images may be mutually canceled. In contrast, MSE calculates the counting error for each image independently. The performances of the three network architectures in people-counting are shown in Table 6. The main reason is that with the top-down module and the proposed prediction module, our method was able to generate features of much higher quality for classification and regression, hence reducing the number of false alarms and missing detections significantly in various challenging situations.

## 5. Conclusions

This paper suggests a CNN network to identify whether people are or are not wearing hardhats on construction sites. Thanks to the excellent representation power and learning capabilities of CNNs, the proposed method does not depend on the background subtraction or the full-body people-detection steps as most existing methods do. Instead, only the head region of people is taken as the object of interest and studied, eliminating most of the adverse impacts of occlusion and body deformations while producing robust performance on complex construction sites.

In the proposed network, the MobileNet is adopted as the backbone network for fast multi-scale feature maps generation. To enhance the performance of the network in detecting small objects and dealing with occlusion, the top-down module is leveraged to inject the high-level semantic and contextual information into the low-level feature maps. A residual block is added into the prediction module for further fusing the features from the top-down module, which is beneficial for the final classification and regression. The average precision of the proposed method was 87.4% and 89.4% for no_hardhat and hardhat detection, respectively, with the running speed being 62 FPS. Compared with the well-known object detection networks Faster R-CNN and SSD, our method obtained a higher detection accuracy while requiring a lower computational cost.

Our future work is three-fold. First, the spatial and appearance relationship between the head and the entire body will be studied to resolve more detection ambiguities and hence boost the hardhat-wearing detection performance. Second, the temporal information of videos will be exploited and a detector based on a recurrent neural network will be developed to achieve better performance for hardhat-wearing detection. Third, the proposed detector will be implemented on a field-programmable gate array that can be embedded in surveillance cameras, bringing the method closer to real applications. 

## Figures and Tables

**Table 1:** Performance comparison of different network architectures (bold indicates either best performance or minimum cost).

Architecture	Model Size (M)	Run Time (ms)	${\mathrm{AP}}_{\mathrm{no}\_\mathrm{hardhat}}$ (%)	${\mathrm{AP}}_{\mathrm{hardhat}}$ (%)
Baseline	**13.4**	**12**	82.8	86.0
Baseline+top-down	14.7	14	85.1	88.3
Proposed	18.9	16	**87.4**	**89.4**

**Table 2:** Performance comparison for different prediction modules (bold indicates either best performance or minimum cost).

Prediction Module	Model Size (M)	Run Time (ms)	${\mathrm{AP}}_{\mathrm{no}\_\mathrm{hardhat}}$ (%)	${\mathrm{AP}}_{\mathrm{hardhat}}$ (%)
*a*	22.7	**15**	87.0	88.8
*b*	29.7	17	86.7	89.1
*c*	**18.7**	16	87.2	89.2
Proposed	18.9	16	**87.4**	**89.4**

**Table 3:** Results comparison with other object detection methods (bold indicates either best performance or minimum cost).

Detector	Input Size (pixels)	Model Size (M)	Run Time (ms)	${\mathrm{AP}}_{\mathrm{no}\_\mathrm{hardhat}}$ (%)	${\mathrm{AP}}_{\mathrm{hardhat}}$ (%)
Faster R-CNN	1000×600	607.2	98	85.7	88.6
SSD	300×300	105.1	21	86.3	88.7
Proposed	300×300	**18.7**	**16**	**87.4**	**89.4**

**Table 4:** Results comparison of different network architectures under the occlusion condition (bold indicates either best performance or minimum cost).

Detector	${\mathrm{Prcn}}_{\mathrm{no}\_\mathrm{hardhat}}$	${\mathrm{Rcll}}_{\mathrm{no}\_\mathrm{hardhat}}$	${\mathrm{Acc}}_{\mathrm{no}\_\mathrm{hardhat}}$	${\mathrm{Prcn}}_{\mathrm{hardhat}}$	${\mathrm{Rcll}}_{\mathrm{hardhat}}$	${\mathrm{Acc}}_{\mathrm{hardhat}}$
	(%)	(%)	(%)	(%)	(%)	(%)
Baseline	82.8	65.1	57.3	79.8	58.4	50.9
Baseline+top-down	**89.1**	64.1	59.4	**90.6**	66.4	62.1
Proposed	89.0	**77.6**	**70.8**	86.1	**79.7**	**70.6**

**Table 5:** Results comparison of different network architectures under the low-contrast condition (bold indicates either best performance or minimum cost).

Detector	${\mathrm{Prcn}}_{\mathrm{no}\_\mathrm{hardhat}}$	${\mathrm{Rcll}}_{\mathrm{no}\_\mathrm{hardhat}}$	${\mathrm{Acc}}_{\mathrm{no}\_\mathrm{hardhat}}$	${\mathrm{Prcn}}_{\mathrm{hardhat}}$	${\mathrm{Rcll}}_{\mathrm{hardhat}}$	${\mathrm{Acc}}_{\mathrm{hardhat}}$
	(%)	(%)	(%)	(%)	(%)	(%)
Baseline	86.9	57.0	52.5	**87.3**	45.6	42.8
Baseline+top-down	92.4	57.0	54.5	85.1	58.5	53.3
Proposed	**93.6**	**68.0**	**64.9**	86.4	**76.8**	**68.5**

**Table 6:** People-counting results of different network architectures on the test set. ER: error rate; MSE: mean square error (bold indicates either best performance or minimum cost).

Architecture	#no_hardhat	#hardhat	#total	ER (%)	MSE
Baseline	1698	4344	6042	9.36	1.94
Baseline+top-down	1605	4528	6133	8.00	1.40
Proposed	1752	4766	6518	**2.22**	**0.90**
Ground truth	1803	4863	6666	–	–
